# Wireless Control Combining Myoelectric Signal and Human Body Communication for Wearable Robots

**DOI:** 10.3390/mi13020290

**Published:** 2022-02-12

**Authors:** Taisuke Iguchi, Ikuma Kondo, Jianqing Wang

**Affiliations:** Graduate School of Engineering, Nagoya Institute of Technology, Nagoya 466-8555, Japan; t.iguchi.507@stn.nitech.ac.jp (T.I.); i.kondo.718@stn.nitech.ac.jp (I.K.)

**Keywords:** wearable robot, wireless control, human body communication, communication module

## Abstract

In this study, a communication module based on human body communication was developed to wirelessly control a wearable robot hand based on myoelectric signals. The communication module adopts 10–60 MHz band and an impulse radio multi-pulse position modulation method to achieve low transmission loss and high data rate. A technique to reduce the module size was developed by sharing the myoelectric signal detection electrode and transmitting electrode, and three receiving electrode structures were investigated to improve signal transmission performance. As a result, the developed communication module provides a packet detection rate of 100% and a bit error rate of less than 10${}^{-6}$ up to at least 110 cm along the arm, and a wearable robot hand was demonstrated to be properly controlled based on a human subject’s myoelectric signals.

## 1. Introduction

Wearable robots are attracting a great deal of attention in today’s aging society with a declining birthrate. Controlling wearable robots according to the human will is expected in fields such as industrial robots and disaster relief robots. A wearable robot hand can replace real human hand to perform more complex and precise tasks. It can also be expected to work as a third human hand. For patients with severe disabilities in limb movement or language function, a wearable robot hand such as electric artificial hand is also very useful if it can be controlled by human will. The most popular method to control a wearable robot hand is to use myoelectric signals. It usually consists of a sensor unit which detects the myoelectric signals, a controller unit which analyzes the signals and generates a pulse width modulation signal necessary for motor control, and a motor drive unit to drive the robot hand [1,2,3,4,5,6,7]. A distance usually exists between the myoelectric signal sensor unit and the motor drive unit, so that some wires have to be used for signal connection between the two units. These wires not only cause inconvenience during use, but also often act as antennas to cause electromagnetic compatibility (EMC) problems [8,9]. Therefore, it is expected to replace the wires with wireless technology [10,11].

**Figure 1:**
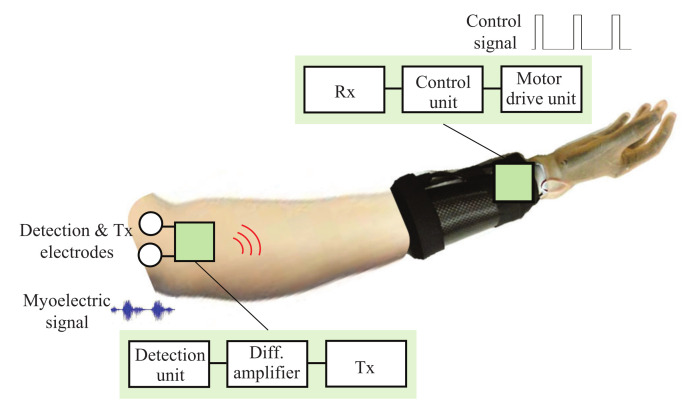
Concept of a wearable robot hand controlled using myoelectric signals.

Figure 1 illustrates the concept of a wearable robot hand controlled using myoelectric signals. Both the sensor unit and the motor drive unit are on or near the human body. Detection of myoelectric signals in the sensor unit typically uses multiple pairs of electrodes that come into contact with the skin surface of the arm. Technologies available for wireless transmission between the sensor unit and the motor drive unit include Bluetooth, WiFi, and ZigBee at 2.4 GHz band, ultra wide band (UWB) technology using 3.1–10.6 GHz [12], and human body communication (HBC) [13,14]. The technologies are often used in wireless body area netwoeks (WBANs) [15]. WBAN is a network which requires a number of sensors to be connected. To manage the transmission and reception of signals from many sensors, complex medium access control (MAC) layer protocols are required [16,17,18,19,20]. However, the purpose in this study is to wirelessly connect a myoelectric signal sensor and a robot hand controller. This is a point-to-point communication and does not require network protocols. As analyzed in [10], for frequencies above hundreds of MHz, the mechanism of wireless transmission on the human body is based on radiated field components. However, HBC typically uses a few MHz to a few tens of MHz. Since the human body looks like a conductive medium at these frequencies, the mechanism of wireless transmission on the human body is based on electric field coupling (or capacitive coupling). It is a different mechanism of transmission from 2.4 GHz band or UWB. Instead of wireless transmission at 2.4 GHz band or UWB, which uses an antenna to radiate radio-frequency signals, HBC uses a transmitting electrode to apply signal to the human body and a receiving electrode to detect the signal from the human body according to its electric field coupling mechanism.

Table 1 compares the superiority and inferiority of the three technologies for wireless transmission on the human body. Bluetooth is used as a representative of the 2.4 GHz band. As can be seen, HBC shows four advantages over Bluetooth and UWB: (1) HBC has a much smaller transmission loss than Bluetooth and UWB due to the frequency-dependent lossy dielectric properties of the human body [10]. (2) HBC does not radiate radio-frequency signal to the outside, so, it has higher security than Bluetooth and UWB. The signal transmission in HBC is mainly along the body surface due to its electric field coupling mechanism. (3) Because of the wide bandwidth, HBC can provide faster transmission speeds than Bluetooth, although it is not as fast as UWB. (4) HBC is advantageous for miniaturizing the transmitter (Tx) by using one electrode for both detection and transmission of myoelectric signals (as described in Section 3). Therefore, HBC is very well suited for wearable robot control compared to other short-range wireless communication technologies.

In this paper, focusing on the use of myoelectric signals for wearable robot control, we present a wireless control method for wearable robots which combines myoelectric signal detection and HBC. Section 2 describes the developed HBC module structure. Section 3 describes how to share the detection electrode and transmitting electrode for Tx module miniaturization, and Section 4 describes the electrode structure suitable for the receiver (Rx) module. Section 5 describes the communication performance of the HBC module and experimental result for driving a robot hand based on myoelectric signals. Lastly, Section 6 concludes this paper.

## 2. Tx and Rx Module Structure

**Figure 2:**
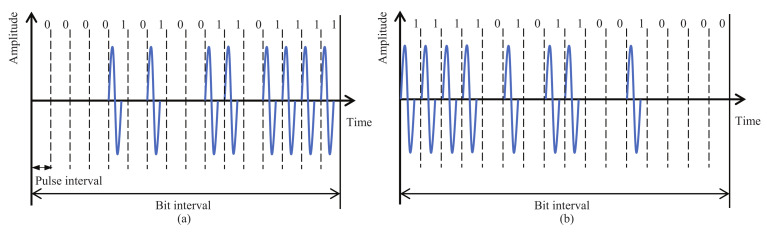
Conceptual time waveform of IR-MPPM with a spreading rate of 16. (**a**) Information bit “0”. (**b**) Information bit “1”.

**Figure 3:**
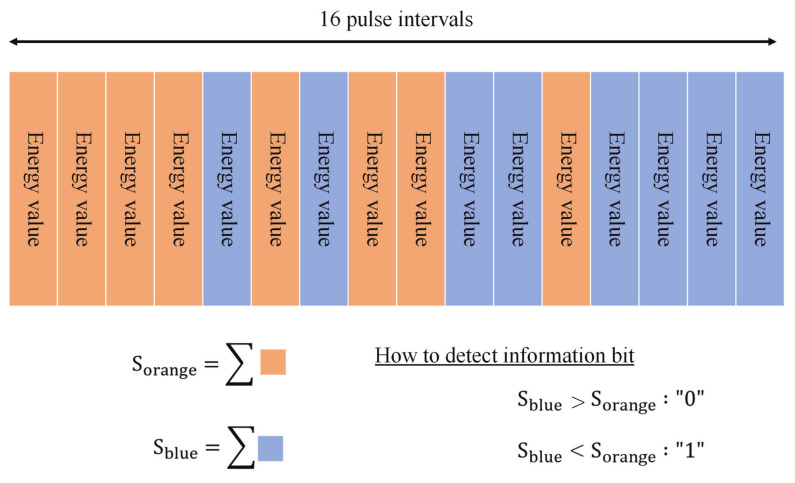
How to demodulate information bit by energy detection.

The basic structure of the HBC module for wearable robot control has already been reported in [11,21]. The current version aims for further miniaturization and longer transmission distance. In order to make effective use of the wide bandwidth, we adopted a wide band impulse radio (IR) scheme, which directly transmits pulse signals without using a carrier. Information bits are represented by the temporal position of multiple pulses, called multi-pulse position modulation (MPPM). This modulation scheme contributes to a simple circuit structure and low power consumption. To improve the communication performance for achieving longer transmission distances, IR-MPPM spreads one information bit over multiple pulses. Figure 2 shows the time waveform with a spreading rate of 16, where 16 pulses are used to represent one information bit. This technique can effectively improve the energy per bit to noise power spectral density ratio and bit error rate (BER) performance. By changing the spreading rate, the data rate can be changed according to the communication environment and transmission speed requirements. To demodulate the IP-MPPM signal, we adopted an energy detection method by comparing the total energy of the pulses in the orange period and the blue period, as shown in Figure 3. This method does not require a threshold to determine “0” and “1”.

**Figure 4:**
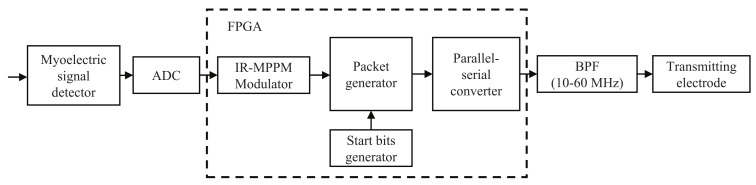
Block diagram of the Tx module implemented in a field programmable gate array (FPGA).

Figure 4 shows the block diagram of the Tx module, and Table 2 shows the specifications of the Tx module. First, an analog to digital converter (ADC) digitizes the myoelectric signal from the detector. The modulator then modulates the digitized myoelectric signal by the IR-MPPM. The packet generator generates the packets, and each packet consists of a 4-bit long start bit and a 36-bit long payload. The packet rate is set to 2 kHz, which is the same as the sampling rate of the ADC. The parallel-serial converter outputs pulses at a pulse rate of 20 Mp/s. Since the spreading rate is 16, the data rate will be 1.25 Mb/s. The output pulses pass through the spectrally shaped BPF and are then transmitted from the transmitting electrode.

**Figure 5:**
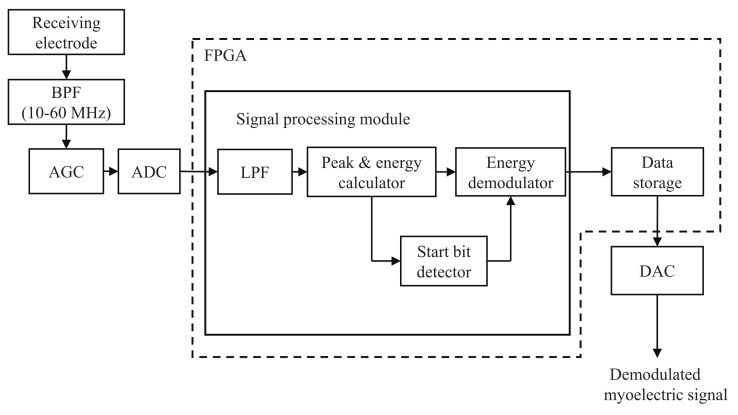
Block diagram of the Rx module.

Figure 5 shows the block diagram of the Rx module with a physical size of 48 × 35 mm${}^{2}$. The Rx module consists of a BPF, an automatic gain controller (AGC), an ADC, a signal processing module, a data storage, and a digital to analog converter (DAC). First, the received signal is filtered by the BPF and amplified by the AGC to an appropriate level. The ADC then digitizes the amplified signal at a sampling frequency of 200 MHz. The signal processing module extracts the envelope and calculate the peak and energy values. The peak and energy values are used to detect the start bits and demodulate the information bits, respectively. Finally, the demodulated information bits are stored in the data storage, and output from the DAC is taken for use in the wireless control of robot hand.

## 3. Transmitting Electrode Structure

Since the transmission of myoelectric signals uses HBC technology, the structure of the transmitting and receiving electrodes plays an important role. This section focuses on sharing the myoelectric signal detection electrode as the transmitting electrode.

**Figure 6:**
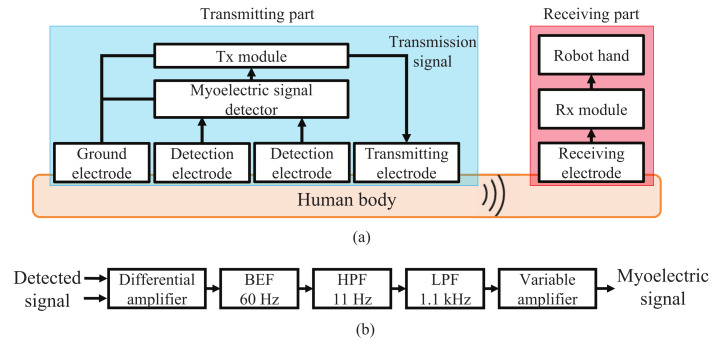
Conventional myoelectric signal transmission using HBC. (**a**) Block diagram. (**b**) Internal configuration of myoelectric signal detector.

Figure 6a shows a block diagram of myoelectric signal transmission using HBC for wearable robot control. Figure 6b shows the internal configuration of the myoelectric signal detector consisting of a differential amplifier, a band elimination filter (BEF), a high pass filter (HPF), a low pass filter (LPF), and a variable amplifier. The BEF is used to block 50 or 60 Hz noise from the commercial power supply, and the HPF and LPF are used to remove artifacts and unnecessary frequency components caused by breathing, body movement and others. The voltage level of myoelectric signals ranges from tens of μV to several mV. In contrast, the voltage level of common mode (CM) noise induced in the human body by an external electromagnetic field such as a commercial power supply or a transmission signal applied to the human body from the Tx module is about 100 to 1000 times that of the myoelectric signals. Therefore, usual detector uses three electrodes consisting of two signal detection electrodes and a ground electrode, and inputs the potential difference between the two detection electrodes to the differential amplifier to detect the myoelectric signal and cancel the CM noise. However, due to the difference in contact impedance between the two detection electrodes with the human body, the CM noise cannot be completely canceled and part of the CM noise is converted to differential mode interference voltage.

**Figure 7:**
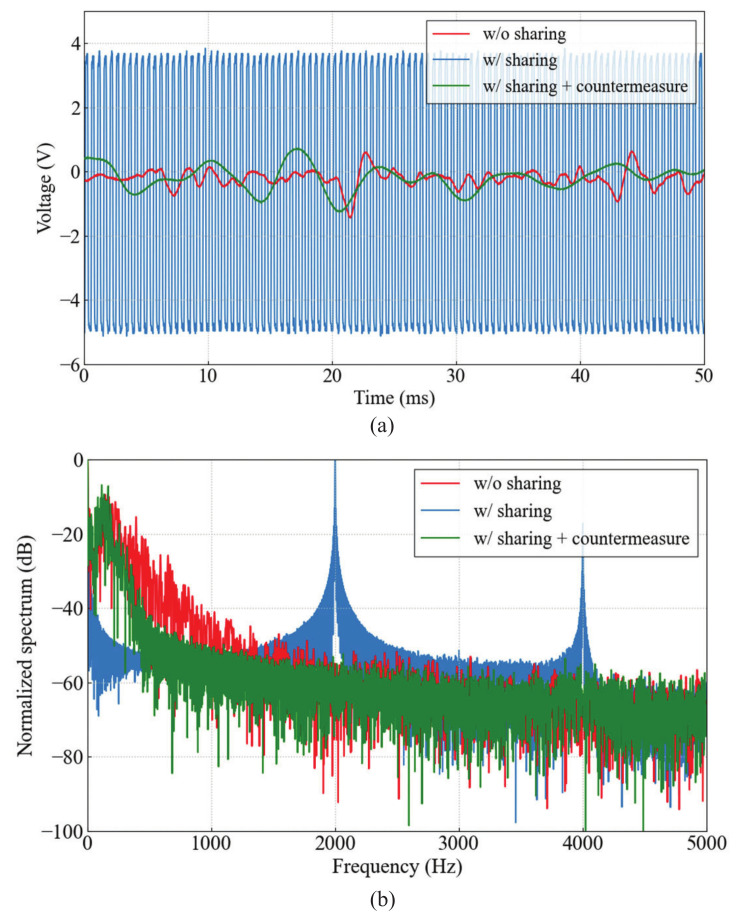
(**a**) Time waveform and (**b**) frequency spectrum of output from the myoelectric signal detector. The waveforms w/o sharing and w/sharing + countermeasure are a myoelectric signal at different times.

Since the myoelectric signal is detected and transmitted in different time slots, the myoelectric signal can be detected correctly without being affected by the CM noise generated by signal transmission. This allows one of the two signal detection electrodes to be used as a transmission electrode, i.e., the signal detection electrode and the transmitting electrode can be shared. However, the voltage levels of the transmission signal superimposed on the shared and non-shared detection electrodes will be significantly different. This causes the transmission signal to be converted into an interference voltage without being canceled by the differential amplifier. The interference voltage is band-limited by the filters and amplified by the variable amplifier. Figure 7 shows the time waveform and frequency spectrum of the output from the myoelectric signal detector when one of the two detection electrodes is used as the transmitting electrode. The interference voltage is a pulse repeated at 2 kHz, which is the same as the packet rate of the transmission signal, and prevents the detection of myoelectric signal in the range of 10 to 300 Hz.

**Figure 8:**
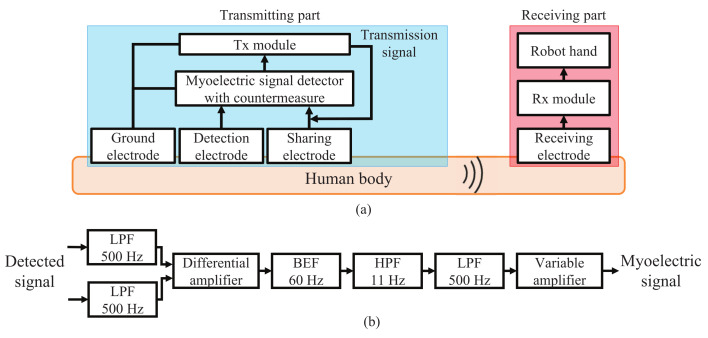
Myoelectric signal transmission using HBC which shares the signal detection electrode and transmitting electrode. (**a**) Block diagram. (**b**) Internal configuration of myoelectric signal detector.

In order to use one of the two detection electrodes as the transmitting electrode, interference countermeasures are required. We added an LPF with a cut-off frequency of 500 Hz to each of two inputs of the differential amplifier. This cuts off the transmission signal in 10–60 MHz band without interfering with the detection of the myoelectric signals up to 300 Hz. Figure 8 shows the block diagram of the myoelectric signal transmission using HBC for wearable robot control after applying the interference countermeasure. As can be seen from the time waveform and frequency spectrum of the output from the myoelectric signal detector, which is also shown in Figure 7, the interference voltage is reduced by about 50 dB at maximum due to the interference countermeasure. As a result, the detection of myoelectric signals up to 300 Hz is achieved by sharing one of the detection electrodes as the transmission electrode. This transmitting electrode structure contributes to the overall miniaturization of the myoelectric signal detector and the Tx module.

## 4. Receiving Electrode Structure

HBC is roughly divided into two types, the current type and the electric field type. In the current type, a pair of electrodes generates a weak current to flow through the human body, and the signal is transmitted at that current. On the other hand, the electric field type uses changes in the electric field generated on the surface of the human body to transmit a signal. The signal is applied and detected by capacitive coupling with the human body. Different transmission types require different electrode structure.

**Figure 9:**
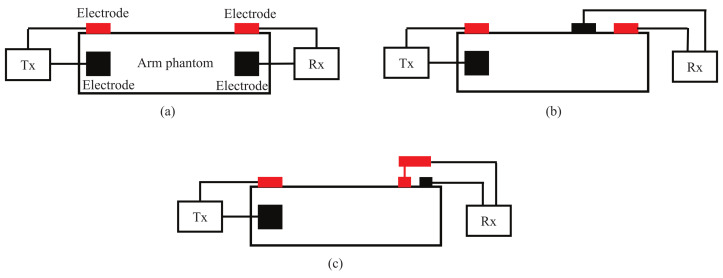
Three receiving electrode structures. The red represents the signal (hot) electrode, and the black represents the ground (cold) electrode. (**a**) Structure A. (**b**) Structure B. (**c**) Structure C.

In this study, as described in the previous section, the structure of the transmitting electrode is used as the current type to share one of the detection electrodes as the transmitting electrodes. For the receiving electrodes, Figure 9 shows three structures. Structures A and B can be considered current type, and structure C can be considered electric field type.

**Figure 10:**
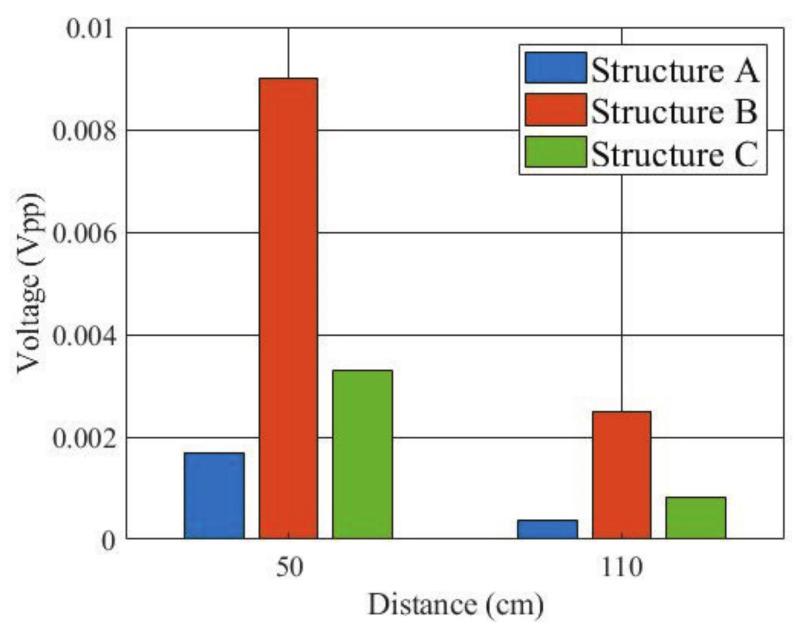
Simulated peak-to-peak values of received voltage for the three receiving electrode structures.

In order to quantitatively evaluate the performance of the three types of receiving electrode structures, finite difference time domain (FDTD) simulation using a numerical arm phantom was first performed. The numerical phantom had the same dielectric properties as the gel phantom used in experiment. The length of the phantom was 50 cm or 110 cm. A Gauss pulse with −10 dB bandwidth of 10 to 60 MHz and peak voltage of 10 V was applied to the transmitting electrode, and the received signal was detected on the receiving electrode. Figure 10 shows the peak-to-peak values of the received voltage. The received voltage of structure B was the highest of the three structures at both distances.

**Figure 11:**
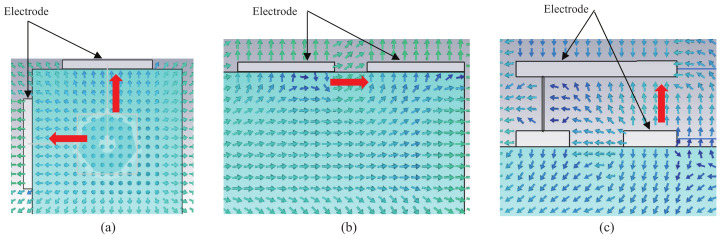
Comparison of electric field distributions at 40 MHz for the three receiving electrode structures. (**a**) Structure A. (**b**) Structure B. (**c**) Structure C.

In addition, Figure 11 shows the simulated electric field distributions near the three receiving electrode structures at 40 MHz, in which the blue parts represent the numerical arm phantom. Figure 11a is a cross-sectional view of the arm phantom, and Figure 11b,c are longitudinal cross-sectional views of the arm phantom. The red arrows indicate the direction of the electric field between the electrodes. As can be seen, unlike other structures, the structure A has the worst performance because the electric fields between the receiving electrodes do not point in the same direction and the field levels are also weak.

**Figure 12:**
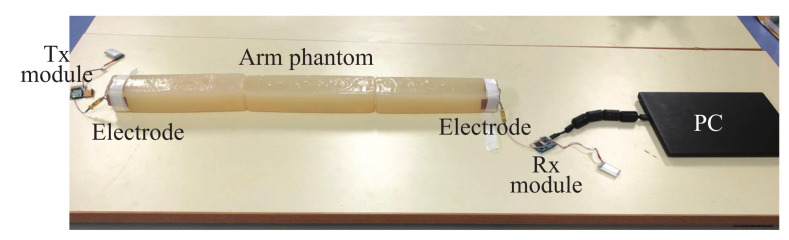
View of signal transmission experiment for investigating the three electrode structures.

**Figure 13:**
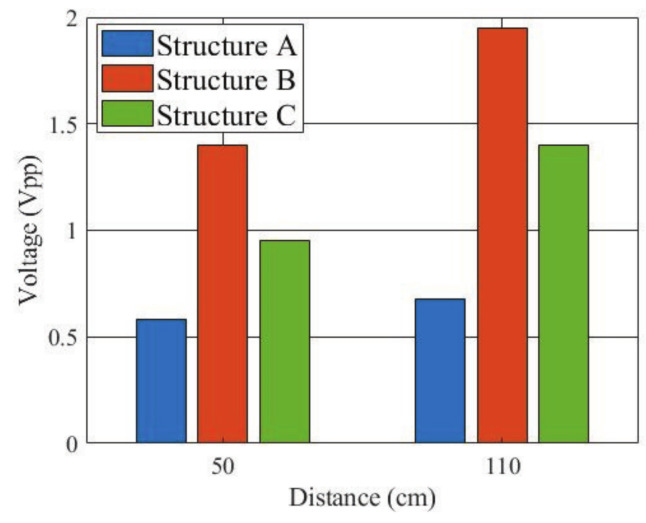
Measured peak-to-peak values of received voltage for the three receiving electrode structures.

Moreover, an experiment was also conducted to verify the simulation result. Figure 12 shows the signal transmission experiment. The three electrode structures were manufactured and connected to the Tx and Rx modules, respectively. The arm was modeled using a gel phantom [22] with a length of 50 cm or 110 cm. The dielectric properties of the phantom were close to the average of the arm [23]. The transmitted signal was an IR-MPPM signal and the received pulse voltage was detected at the AGC output of the Rx module. After the ADC, it was sent to a PC via a USB cable. Figure 13 shows the measured received peak-to-peak voltage. Again, the received voltage of the structure B was the highest at both distances, and the relative magnitude between the three types was the same as in the simulation. The reason why the received voltage at 110 cm was higher than the received voltage at 50 cm is that the AGC operated with different amplification factors at 50 cm and 110 cm.

## 5. Performance Evaluation

**Figure 14:**
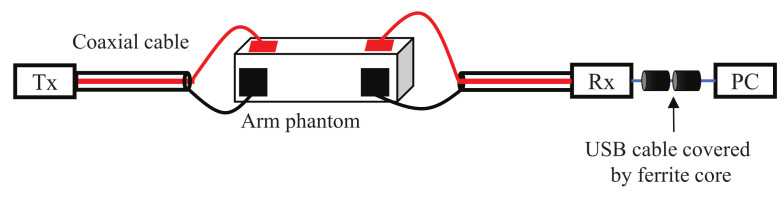
Block diagram of PDR and BER measurements.

This section first reports the packet detection rate (PDR) and BER measurement results for communication performance evaluation. The measurement was conducted in a normal room using a gel phantom simulating a human arm. Table 3 shows the measurement conditions, and Figure 14 shows the conceptual diagram of the PDR and BER measurements. All the three receiving electrode structures were used for comparison. The transmission distance on the arm gel phantom was changed to 20 cm, 50 cm, 80 cm, and 110 cm, respectively. Since the myoelectric signal is detected at the arm and the length of a human arm usually does not exceed 100 cm, the length of the arm phantom was set to 110 cm and the verification experiment was limited to 110 cm. The packets transmitted from the Tx module were 10${}^{5}$ packets corresponding to 3.6 × 10${}^{6}$ bits. The received signals were demodulated by the Rx module and the demodulated information bits were sent to a PC via a USB cable to calculate the PDR and BER.

**Figure 15:**
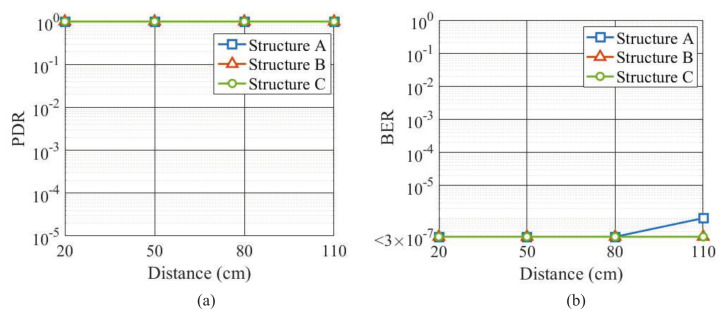
(**a**) Measured PDR versus distance. (**b**) Measured BER versus distance.

Figure 15 shows the PDR and BER measurement results as a function of transmission distance. The Rx module consists of an AGC. The longer the transmission distance, the higher the gain of the amplifier and the more noise power it produces. Therefore, the longer distance corresponds to larger noise power and lower signal to noise power ratio (SNR). Since it is difficult to obtain the SNR by separating the signal power and noise power in the Rx module, the PDR and BER performances are shown versus the transmission distance. It is found that the PDR is the same for the three receiving electrode structures, but the BER of the structures B and C is better than the structure A. This is consistent with the result in the previous section. At all distances investigated, the PDR achieves 100% and the BER is less than 10${}^{-6}$ for the three electrode structures. Such PDR and BER levels are well acceptable at the physical layer of wireless communication. However, if we assume to transmit a brain wave signal from the head to the hand, the transmission distance can be longer than 110 cm, and the PDR will deteriorate according to our preliminary measurement result [24]. In addition, the main noise in the measurement is Gaussian thermal noise from the amplifier. In actual use, impulse noise such as electrostatic discharge from the human body itself may also occur [25]. In that case, achieving a PDR of 100% is not easy because the peak voltage of impulse noise is too large.

**Figure 16:**
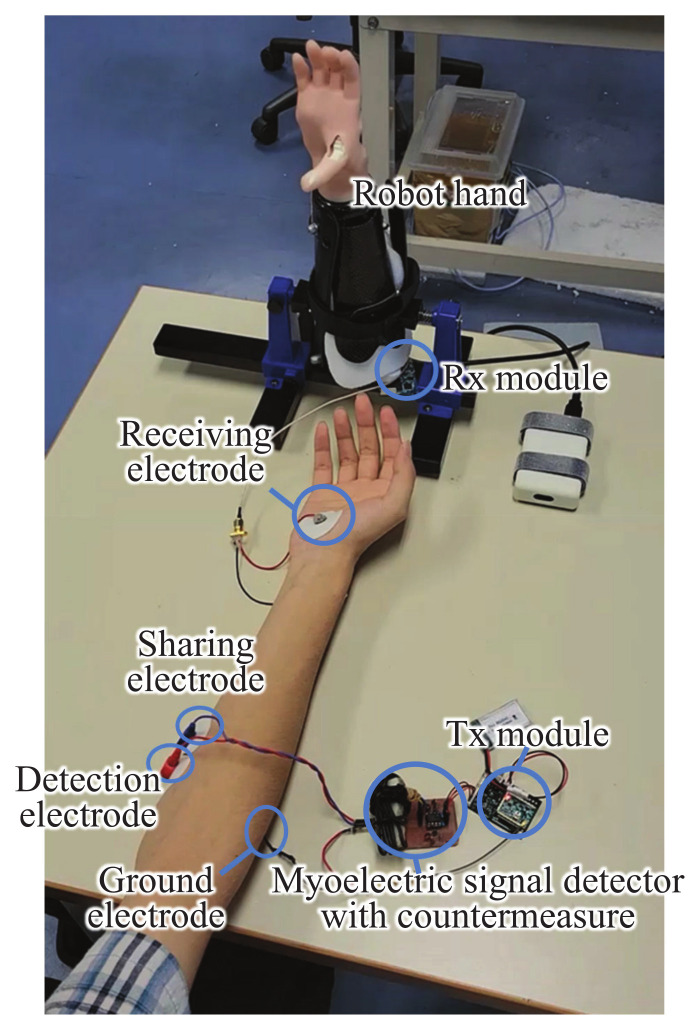
View of experiment using HBC to transmit myoelectric signal for wearable robot hand control.

Moreover, an experiment to operate a wearable robot hand was conducted using the developed HBC modules for wireless control. As discussed, for the receiving electrode structure, any of the three structures can be used, but the structure B provides the best performance. In this experiment, however, the structure A was used to consider the worst case. Figure 16 shows the experimental view in a normal room. The myoelectric signal was detected from the human subject’s shallow finger flexors and sent to the robot hand for wireless control. The human subject was asked to perform two actions. One is to hold the hand for five seconds, and the other is to open the hand for five seconds. The two actions were alternately repeated five times to see whether the robot hand acts according to the human subject’s hand actions. Table 4 summarizes the success rate of wireless control of the wearable robot hand using the developed HBC module. The result shows a success rate of 100%, which demonstrates that the developed Tx and Rx modules are suitable for controlling the robot hand using myoelectric signals.

## 6. Conclusions

An HBC-based communication module has been developed to wirelessly control the robot hands using myoelectric signals. The use of the 10–60 MHz band has contributed to low transmission loss and high data rate (typically 1.25 Mb/s, up to 20 Mb/s). For transmitting electrode, a technique has been developed to share the detection electrode and transmitting electrode to reduce the Tx module size. Three receiving electrode structures have also been investigated to improve the transmission performance. As a result, up to a communication distance of at least 110 cm along the arm, the PDR achieves 100% and the BER is less than 10${}^{-6}$ for any of the three electrode structures. This performance is well acceptable at the physical layer of wireless communication. Moreover, the experiment to operate a wearable robot hand has also shown that the developed Tx and Rx modules properly control the robot hand based on the human subject’s myoelectric signals.

The challenge for future research is to extend this technology to wearable robot control using brain waves.

## Figures and Tables

**Table 1:** Comparison of Bluetooth, UWB, and HBC for wireless transmission on the human body.

	Bluetooth	UWB	HBC
Frequency	2.4 GHz	3.1–10.6 GHz	10–60 MHz
Bandwidth	∼1 MHz	∼500 MHz	∼50 MHz
Transmission speed	Not fast	Very fast	Fast
Transmission loss	Large	Very large	Small
Transmission mechanism	Radiation	Radiation	Electric field coupling
Antenna	Need	Need	Not need
Sharing of electrodes	Not possible	Not possible	Possible

**Table 2:** Specifications of the Tx module.

Frequency band	10–60 MHz
Modulation method	Impulse radio-Multi-pulse position modulation
Pulse rate	20 Mp/s
Date rate	1.25 Mb/s
Spreading rate	16
Output power	−15 dBm/MHz
Power supply	3.3 V/2.7 V
Consumption current	17 mA
Physical size	44 × 33 mm${}^{2}$ (including myoelectric signal detection unit)

**Table 3:** PDR and BER measurement conditions.

Transmission distances	20 cm, 50 cm, 80 cm, 110 cm
Transmitted packets	10${}^{5}$ packets
Transmitted bits	3.6 × 10${}^{6}$ bits

**Table 4:** Success rate of wireless control of robot hand.

Keep the hand grasping	100%
Keep the hand opening	100%

## References

[1] Chen F., Li Z., Chen C.L.P., Agaian S. (2018). Introduction to the special issue on human cooperative wearable robotic systems. IEEE Robot. Autom. Lett..

[2] Seki T., Nakamura T., Kato R., Morishita S., Yokoi H. (2014). Development of five-finger multi-DoF myoelectric hands with a power allocation mechanism. J. Mech. Eng. Autom..

[3] Randazzo L., Iturrate I., Perdikis S., Millan J.d.R. (2018). mano: A wearable hand exoskeleton for activities of daily living and neurorehabilitation. IEEE Robot. Autom. Lett..

[4] Chen M., Zhou J., Tao G., Yang J., Hu L. (2018). Wearable affective robot. IEEE Access.

[5] Wang Y., Tian Y., She H., Jiang Y., Yokoi H., Liu Y. (2022). Design of an effective prosthetic hand system for adaptive grasping with the control of myoelectric pattern recognition approach. Micromachines.

[6] Islam M.R., Assad-Uz-Zaman M., Brahmi B., Bouteraa Y., Wang I., Rahman M.H. (2021). Design and development of an upper limb rehabilitative robot with dual functionality. Micromachines.

[7] Triwiyanto T., Caesarendra W., Purnomo M.H., Sułowicz M., Wisana I.D.G.H., Titisari D., Lamidi L., Rismayani R. (2022). Embedded machine learning using a multi-thread algorithm on a Raspberry Pi platform to improve prosthetic hand performance. Micromachines.

[8] Morinaga Y., Nagai K., Wang J., Anzai D. (2020). Impact of electrostatic discharge on wearable robotic hand and improvement of immunity performance by wireless control. IEEE Electromagn. Compat. Mag..

[9] Wang J., Nakaya R., Sato K., Anzai D., Fujiwara O., Amemiya F. (2019). Development of an immunity test system with a pseudo biosignal generator for wearable devices and application to the ESD test of an artificial hand. IEEE Trans. Electromagn. Compat..

[10] Wang J., Wang Q. (2012). Body Area Communications.

[11] Wang J. (2020). Wide band human body communication technology for wearable and implantable robot control. IEICE Trans. Commun..

[12] (2002). Revision of Part 15 of the Commission’s Rules Regarding Ultra-Wideband Transmission System: First Report and Order.

[13] Zimmerman T.G. (1996). Personal area networks: Near-field intrabody communications. IBM Syst. J..

[14] Baldus H., Corroy S., Fazzi A., Klabunde K., Schenk T. (2009). Human-centric connectivity enabled by body-coupled communications. IEEE Commun. Mag..

[15] (2012). IEEE Standard for Local and Metropolitan Area Network-Part 15.6: Wireless Body Area Networks.

[16] Monton E., Hernandez J.F., Blasco J.M., Herve T., Micallef J., Grech I., Brincat A., Traver V. (2008). Body area network for wireless patient monitoring. IET Commun..

[17] Bayrakdar M.E. (2019). Priority based health data monitoring with IEEE 802.11af technology in wireless medical sensor networks. Med. Biol. Eng. Comput..

[18] Cicioğlu M., Çalhan A. (2019). SDN-based wireless body area network routing algorithm for healthcare architecture. ETRI J..

[19] Cicioğlu M., Çalhan A. (2020). Energy-efficient and SDN-enabled routing algorithm for wireless body area network. Comput. Commun..

[20] Bayrakdar M.E. Fuzzy Logic based coordinator node selection approach in wireless medical sensor networks. Proceedings of the 2019 4th International Conference on Computer Science and Engineering (UBMK).

[21] Ando H., Murase Y., Anzai D., Wang J. (2019). Wireless control of robotic artificial hand using myoelectric signal based on wideband human body communication. IEEE Access.

[22] Ito K., Kawai H., Saito K. (2002). State of the art and future prospects of biological tissue-equivalent phantoms. Trans. IEICE.

[23] Gabriel G. (1996). Compilation of the dielectric properties of body tissues at RF and microwave frequencies. Brooks Air Force.

[24] Liao W., Muramatsu K., Wang J. (2021). Path loss analysis and transceiver development for human body communication-based signal transmission for wearable robot contro. IEEE Access.

[25] Liao W., Nagai K., Wang J. (2020). An evaluation method of electromagnetic interference on bio-sensor used for wearable robot control. IEEE Trans. Electromagn. Compat..

